# Healthcare Resource Utilisation and Cost of Obesity and Related Complications in the United States: A Systematic Literature Review

**DOI:** 10.1111/dom.70602

**Published:** 2026-03-09

**Authors:** Jaime P. Almandoz, Jamy D. Ard, Chellse Gazda, Shenelle Edwards‐Hampton

**Affiliations:** ^1^ University of Texas Southwestern Medical Center Dallas Texas USA; ^2^ Wake Forest School of Medicine Winston‐Salem North Carolina USA

**Keywords:** cost‐effectiveness, health economics, obesity care, obesity therapy, real‐world evidence, systematic review

## Abstract

**Aim:**

Obesity is an increasingly prevalent, chronic, heterogeneous disease, associated with a variety of obesity‐related complications (ORCs). This systematic literature review assessed US healthcare resource utilisation (HCRU) and costs (direct and indirect) for people living with obesity (PwO) or overweight and ORCs.

**Methods:**

A search was conducted in January 2024 of studies published from 2019 to present reporting HCRU or costs in adults living with obesity (body mass index [BMI] ≥ 30.0 kg/m^2^) with/without ORCs or overweight (BMI ≥ 27.0 kg/m^2^) with ORCs.

**Results:**

Of 6862 studies identified, 60 met inclusion criteria; 23 reported costs/HCRU in obesity alone and 37 reported obesity and ORCs. In general, PwO with/without ORCs reported higher direct and indirect healthcare costs and HCRU than those without obesity. The highest per‐person‐per‐year healthcare costs were for PwO and chronic kidney disease, heart failure, or type 2 diabetes. PwO and ORCs had up to 23% higher hospitalisation costs and longer hospital lengths of stay (5.2–25.0 vs. 4.9–20.0 days) than those with the same conditions without obesity or with lower BMI. Weight loss resulted in decreases in annual medical expenditure (up to $263 per person per month in people who lost 10%–20% of their bodyweight).

**Conclusions:**

The rising rates of obesity and overweight with/without ORCs create a burden on individuals and the US healthcare system, highlighting a need for more effective prevention and treatments to improve health and reduce associated healthcare costs.

## 1 Introduction

Obesity is an increasingly prevalent, heterogeneous disease of energy dysregulation caused by genetic, environmental and behavioural factors. The World Health Organisation defines overweight as a body mass index (BMI) ≥ 25 kg/m^2^ and obesity as ≥ 30 kg/m^2^ [1]. Obesity is subdivided into classes I (BMI 30 to < 35 kg/m^2^), II (35 to < 40 kg/m^2^) and III (≥ 40 kg/m^2^) [2]. Recognition of obesity as a disease has important implications for public health policy, research and reducing stigma.

In 2019, the total economic impact of overweight/obesity in the US was almost $706 billion (43% [$304 billion] direct costs; 57% [$401 billion] indirect costs), of which premature mortality accounted for almost $290 billion [3]. The US economic burden of overweight/obesity is predicted to rise to $1005 billion by 2030 (equivalent to $2875 per person and 3.78% of US GDP) [3]. Moreover, people living with obesity (PwO) or overweight have an increased risk of cardiovascular (CV) diseases, type 2 diabetes (T2D), chronic kidney disease (CKD), musculoskeletal diseases, metabolic dysfunction‐associated steatotic liver disease (MASLD) and some cancers [4, 5]. These obesity‐related complications (ORCs) are associated with an economic burden [6] arising from direct and indirect costs [3].

Given the profound effects of overweight/obesity on society, regular updated data pertaining to the US population are needed. A recent systematic literature review (SLR) on the economic burden of obesity included 19 studies, but only one reported US data [7]. A 2016 SLR reported on medical costs of obesity in the US, but did not mention ORCs [8]. A more recent SLR reported direct costs associated with obesity, ORCs and weight loss in the US, but omitted indirect costs and healthcare resource utilisation (HCRU) [9]. To address these evidence gaps, we conducted a broad and comprehensive narrative synthesis of recently published literature to assess data on US HCRU and costs (direct/indirect) for adults living with obesity (BMI ≥ 30.0 kg/m^2^) with/without ORCs, or having overweight (BMI ≥ 27.0 kg/m^2^) with ORCs.

## 2 Methods

### 2.1 Search Strategy and Selection Criteria

This SLR followed the PRISMA 2020 statement, and PRISMA protocol guidelines. The protocol was prospectively registered with PROSPERO (CRD42024491292). One protocol amendment was made to ensure that included studies were focused on the research question.

To identify controlled trials, real‐world evidence and economic evaluations, Embase, MEDLINE, the Cochrane Central Register of Controlled Trials and the Cochrane Database of Systematic Reviews were searched for the period 1 January 2019–26 January 2024 using MeSH or EMTREE terms and keywords relevant to the population and outcomes of interest. Detailed search strings are provided in Tables S1–S4. The searches were restricted to the publication period to capture the most recent and relevant evidence on the economic burden of obesity and ORCs, as previous reviews either had limited US studies [7], focused on direct costs only [8, 9], or did not assess ORCs [7]. Furthermore, only peer‐reviewed articles were considered, with no searches performed to capture grey or unpublished data.

Studies were included if the population of interest was US adults with a BMI ≥ 30.0 kg/m^2^ with/without ORCs, or BMI ≥ 27.0 kg/m^2^ with ORCs. The ORCs included CV complications (CV disease, heart failure, stroke), diabetes (mixed or type 2 only), osteoarthritis (OA), sleep apnea and liver‐related, pancreas‐related and mental health‐related complications; studies reporting multiple complications were included. Although obesity is a global concern, we limited the SLR to the US because it has a unique healthcare system and represents the third largest population with obesity globally [10].

Studies were excluded if conducted outside the US, not originally published in English, or focused on paediatric populations or adults with a BMI < 27.0 or ≥ 27.0 to < 30.0 kg/m^2^ without ORCs. Case reports and congress abstracts were also excluded. Although reviews were excluded, reference lists from relevant reviews were screened for primary sources.

### 2.2 Study Screening, Quality Assessment and Data Extraction

After removing duplicates, one reviewer screened titles/abstracts for eligibility, with a 20% quality check performed by a second reviewer. The same process was followed for full‐text screening. Per PRISMA guidelines, discrepancies during each round of screening were resolved through discussion, with arbitration by a third (senior) reviewer if needed. Full‐text publications were assessed for quality and risk of bias using the checklist from Molinier et al. [11], adapted to cost of illness (Table S5). Data extraction included HCRU, direct/indirect costs and cost drivers. Outcomes were reported descriptively as means (with cost years), ranges and comparative statistics, where available. Costs were presented as reported, without standardisation or inflation adjustment, and as such were not intended to be compared across studies. Instead, overall trends in costs and HCRU for PwO versus those without are presented as reported in the studies included. No meta‐analysis was performed.

## 3 Results

**FIGURE 1:**
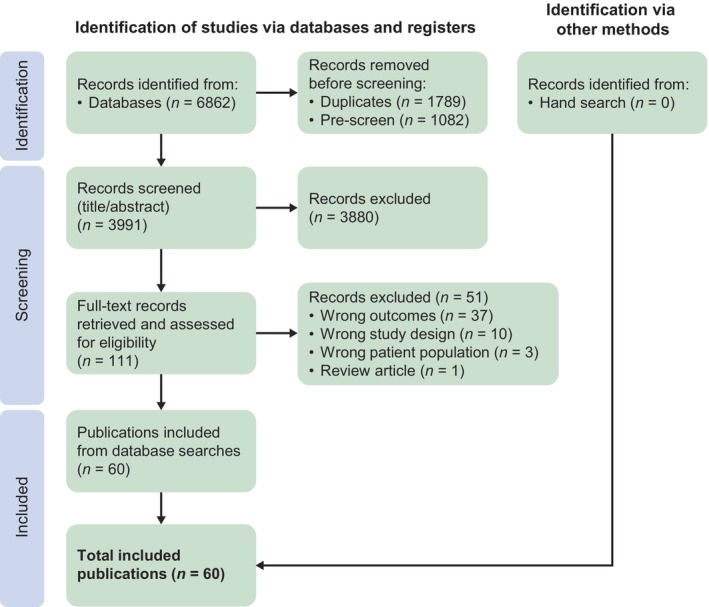
PRISMA flow diagram. PRISMA, Preferred Reporting Items for Systematic Reviews and Meta‐analyses.

Of the 6862 records identified, 1789 duplicates and 1082 irrelevant records were removed; from the remaining 3991 records, 3880 were excluded. Full‐text publications were retrieved for 111 records, of which 51 were excluded (irrelevant outcomes, incorrect study design, or incorrect patient population), leaving 60 for inclusion (see PRISMA flow diagram, Figure 1).

### 3.1 Study and Patient Characteristics

Details of the 60 included studies are presented in Table 1 [12, 13, 14, 15, 16, 17, 18, 19, 20, 21, 22, 23, 24, 25, 26, 27, 28, 29, 30, 31, 32, 33, 34, 35, 36, 37, 38, 39, 40, 41, 42, 43, 44, 45, 46, 47, 48, 49, 50, 51, 52, 53, 54, 55, 56, 57, 58, 59, 60, 61, 62, 63, 64, 65, 66, 67, 68, 69, 70, 71]. Fifty‐four (90%) were retrospective cohort studies, three (5%) were prospective cohort studies, and three (5%) were cross‐sectional studies. Overall, 46 studies reported HCRU, 35 direct medical costs and eight indirect medical costs associated with obesity. Meaningful between‐study comparisons were difficult, given variations in study design, intervention, comparator groups and outcome measurements.

**TABLE 1:** Characteristics of the 60 studies included in this analysis.

Author and year	Study design	Data source	Sample size	Cost period (year)	Comorbidities included	Sponsor/funding	Outcomes reported		
							Direct costs	Indirect costs	HCRU
Alsuhibani 2022 [12]	Retrospective analysis	FAERS database	15 143	NA (HCRU study)	NR	None			●
Amin 2023 [13]	Retrospective cohort	PINC AI Healthcare Database	67 193	2017	Prophylactic treatment of VTE	Sanofi	●		●
Berger 2021 [14]	Retrospective cohort	IQVIA PharMetrics Plus database	15 635	2019	Nonvalvular atrial fibrillation	Janssen Scientific Affairs LLC	●		●
Berger 2022 [15]	Retrospective cohort	IQVIA PharMetrics Plus database	14 612	2019	VTE	Janssen Scientific Affairs LLC	●		●
Blalock 2023 [16]	Retrospective cohort	National CDW database and VETSNET file	355 229	NA (HCRU study)	NR	Awards from the Veterans Health Administration Office			●
Blaszczak 2020 [17]	Retrospective cohort	NRD	29 412	NR	Acute pancreatitis	NCI and NIDDK	●		●
Boye 2020 [18]	Retrospective cohort	IBM MarketScan Explorys Claims and EMR Data Set	32 012	2018	T2D	Eli Lilly and Company	●		●
Cai 2019 [19]	Retrospective cohort	MDS database	2 323 019	NA (HCRU study)	NR	National Institute on Aging			●
Craig Wood 2021 [20]	Retrospective cohort	Geisinger Health System database	63 567	NA (HCRU study)	NR	Novo Nordisk Inc.			●
Cutshall 2021 [21]	Retrospective cohort	Vizient database	246	NR	VTE	None			●
Dahiya 2023 [22]	Cross‐sectional	NIS database	420 600	NR	Acute pancreatitis	None	●		●
de Souza de Silva 2019 [23]	Prospective cohort	Hospital exercise test	3924	NR	NR	Coordenação de Aperfeiçoamento de Pessoal de Nível Superior (CAPES)/Programa de Doutorado Sanduíche	●		
Ding 2021 [24]	Retrospective cohort	IBM MarketScan Explorys Claims and EMR Data Set	20 488	2018	NR	Novo Nordisk Inc.	●		
Divino 2021 [25]	Retrospective cohort	IQVIA's PharMetrics Plus and AEMR	Ranged from 1275 (HFpEF) and 2260 (PCOS) to 50 962 (GERD)	2017	Multiple	Novo Nordisk Inc.	●		
Doane 2023 [26]	Retrospective cohort	NHWS	1853	NR	Bipolar‐I disorder	Alkermes Inc.	●	●	●
Evans 2023 [27]	Retrospective cohort	IQVIA's PharMetrics Plus and AEMR	28 583	2019	Multiple	Novo Nordisk Inc.	●		●
Garvey 2023 [28]	Retrospective cohort	VHA CDW database	3732	2019	NR	Novo Nordisk Inc.	●		●
Groves 2023 [29]	Retrospective cohort	National Longitudinal Study of Youth—1997 (NLSY97)	8516	NR	NR	None		●	
Harris 2019a [30]	Retrospective cohort	NIS database	31 730	NR	Diabetic foot complications	National Heart, Lung, and Blood Institute to the University of Mississippi Medical Center	●		●
Harris 2019b [31]	Retrospective cohort	NIS database	24 494	2014	Infective endocarditis	National Heart, Lung, and Blood Institute to the University of Mississippi Medical Center	●		●
Hoffman 2020 [32]	Retrospective cohort	NIS database	123 415	2018	Spontaneous intracerebral haemorrhage	None	●		●
Huckfeldt 2020 [33]	Retrospective cohort	Ancillary study to the Look AHEAD RCT	3091	2015	T2D	National Institute on Aging	●		●
Iyengar 2019 [34]	Prospective cohort	University of Michigan WMP	142	NR	NR	National Institutes of Health		●	
Johnston 2020 [35]	Retrospective cohort	Optum Integrated Claims‐Clinical Database	38 050	2015	Osteoarthritis	Johnson & Johnson	●		●
Jones 2020 [36]	Retrospective cohort	Johns Hopkins DataMart Casemix database	1670	NA (HCRU study)	NR	MCIC Vermont			●
Krishnaswami 2019 [37]	Retrospective cohort	Data from weight management program across 21 Kaiser Permanente Northern California	10 693	NA (HCRU study)	NR	Nestlé HealthCare Nutrition Inc.			●
Kumar 2020 [38]	Retrospective cohort	EHR	150	NA (HCRU study)	NR	NIH and NCRR			●
Kumar 2022 [39]	Retrospective cohort	MEPS	210 317	NR	T2D	NR	●		
Laliberté 2021 [40]	Retrospective cohort	IQVIA PharMetrics Plus	10 920	2019	Nonvalvular atrial fibrillation	Janssen Scientific Affairs LLC	●		●
Ludhwani 2019 [41]	Retrospective cohort	NIS database	248 228	NR	Peripheral arterial disease	None	●		●
MacEwan 2021 [42]	Retrospective cohort	NHANES and MEPS	6331	2019	NR	Eli Lilly and Company	●	●	
MacEwan 2023 [43]	Retrospective cohort	NHANES and MEPS	5279	2019	NR	NR	●	●	●
McKinney 2021 [44]	Retrospective cohort	EHR	259	NR	Warfarin‐associated haemorrhage	None			●
Mitchell 2023 [45]	Retrospective cohort	Noom Weight user database, EHRs, and a commercial insurance claims database	57 602	2021	NR	Noom Inc.	●		●
Oladunjoye 2021 [46]	Retrospective cohort	NIS database	194 787	NR	Non‐alcoholic fatty liver disease	None	●		●
Pagidipati 2021 [47]	Retrospective cohort	Duke University Health System EHR system	37 253	NA (HCRU study)	NR	Duke University School of Medicine			●
Patel 2021 [48]	Retrospective cohort	NIS database	21 455 763	2008	Multiple	NR	●		●
Pearson‐Stuttard 2023 [49]	Retrospective cohort	IQVIA's PharMetrics Plus and AEMR	28 583	2019	Multiple	Novo Nordisk A/S	●		
Perales 2020 [50]	Retrospective cohort	EHR	176	NR	Atrial fibrillation or VTE	None			●
Peterson 2019 [51]	Retrospective cohort	Truven MarketScan Commercial Claims and Encounters and Medicare Supplemental databases	7126	2016	Atrial fibrillation	Janssen Scientific Affairs LLC	●		●
Pressman 2023 [52]	Retrospective cohort	Hospital EHRs	1 094 790	NA (HCRU study)	NR	Novo Nordisk Inc.			●
Rajkumar 2023 [53]	Prospective cohort	Patients enrolled in the weight loss program	150	NR	T2D	NR			●
Ramasamy 2019 [54]	Retrospective cohort	Optum Health Reporting and Insights employer claims database	86 221	2018	NR	Novo Nordisk Inc.	●	●	
Ramasamy 2020 [55]	Retrospective cohort	Optum Health Reporting and Insights employer claims database	39 196	NR	NR	Novo Nordisk Inc.	●	●	
Rives‐Sanchez 2020 [56]	Prospective cross‐sectional	Screening program in hospitalised patients	538	NR	Sleep‐disordered breathing	NR			●
Rozen 2022 [57]	Retrospective cohort	NIS database, the HCUP and AHRQ	84 185	NR	Stroke	None			●
Rozjabek 2020 [58]	Prospective cross‐sectional	NHWS	69 742	NR	T2D	Janssen Research and Development LLC		●	
Schuller 2020 [59]	Retrospective cohort	NRD	11 188 940	NR	Acute myocardial infarction	None			●
Shaka 2021 [60]	Retrospective cohort	NIS database	1 032 240	NR	Ischemic cerebrovascular accidents	None	●		●
Spyropoulos 2019 [61]	Retrospective cohort	Truven MarketScan Commercial Claims and Encounters database	5780	2016	VTE	Janssen Scientific Affairs LLC	●		●
Sreenivasan 2021 [62]	Retrospective cohort	NRD	35 555	NR	Cardiogenic shock	None			●
Sudat 2023 [63]	Retrospective cohort	EHR	1737	NA (HCRU study)	NR	NIH and NIDDK			●
Surbhi 2022 [64]	Retrospective cohort	Data from 4 practice‐based research networks	148 499	NR	Multiple	University of Tennessee‐funded 2019 Collaborative Research Network (CORNET) Awards in Health Disparities Research			●
Sutton 2021 [65]	Retrospective cohort	EHR	204	NR	PE	None			●
Temkin‐Greener 2020 [66]	Retrospective cohort	MDS database	565 592	NA (HCRU study)	NR	NIH and National Institute on Aging			●
Thorpe 2021 [67]	Retrospective cohort	MEPS	20 971	NR	Multiple	Livongo Inc.	●		
Watkins 2022 [68]	Retrospective cohort	IBM MarketScan Commercial Claims and Encounters Database	219 971	2019	NR	Novo Nordisk Inc.	●		
Weir 2021 [69]	Retrospective cohort	Optum de‐identified Clinformatics Data Mart Database – DOD database	19 988	2020	Nonvalvular atrial fibrillation and T2D	Janssen Scientific Affairs LLC	●		●
Whitaker 2022 [70]	Retrospective cohort	EHR	288	NR	aPCC for reversal of apixaban and rivaroxaban	None			●
Wu 2019 [71]	Retrospective cohort	MEPS	5140	2015	T2D	None	●		

Abbreviations: AEMR, ambulatory electronic medical records database; AHRQ Agency for Healthcare Research and Quality; aPCC, activated prothrombin complex concentrate; CDW, Corporate Data Warehouse; DOD, date of death; EHR, electronic health records; EMR, electronic medical records; FAERS, FDA Adverse Event Reporting System; GERD, gastroesophageal reflux disease; HCUP, Healthcare Cost and Utilisation Project; HCRU, healthcare resource utilisation; HFpEF, heart failure with preserved ejection fraction; MDS, minimum data set; MEPS, Medical Expenditure Panel Survey; NA, not applicable; NCI, National Cancer Institute; NCRR, National Center for Research Resources; NHANES, National Health and Nutrition Examination Survey; NHWS, National Health and Wellness Survey; NIDDK, National Institute of Diabetes and Digestive and Kidney Diseases; NIH, National Institute of Health; NIS, National Inpatient Sample; NR, not reported; NRD, national readmissions database; PCOS, polycystic ovary syndrome; PE, pulmonary embolism; RCT, randomised controlled trial; T2D, type 2 diabetes; VHA, Veterans Health Administration; VTE, venous thromboembolism; WMP, Weight Management Program.

### 3.2 Risk of Bias

The methodological quality of the studies was rated as high in 24 studies that satisfied ≥ 8 of the 11 quality criteria [14, 15, 18, 24, 25, 26, 27, 28, 33, 35, 36, 39, 40, 42, 43, 48, 49, 51, 54, 55, 61, 68, 69, 71] and as average in 36 studies that met 5–7 of the criteria [12, 13, 16, 17, 19, 20, 21, 22, 23, 29, 30, 31, 32, 34, 37, 38, 41, 44, 45, 46, 47, 50, 52, 53, 56, 57, 58, 59, 60, 62, 63, 64, 65, 66, 67, 70] (Table S5). All reviewed studies provided a satisfactory account of the definition of the illness, epidemiological sources and methods adopted, and all presented results consistently with study methodology.

### 3.3 Studies Reporting Costs and HCRU in PwO (No Focus on ORCs)

Twenty‐three studies reported costs and HCRU associated with obesity without focusing on ORCs (Table 1; Figure S1) [12, 16, 19, 20, 23, 24, 28, 29, 34, 36, 37, 38, 42, 43, 45, 47, 52, 54, 55, 58, 63, 66, 68]. Of these, nine reported direct costs [23, 24, 28, 42, 43, 45, 54, 55, 68], seven indirect costs [29, 34, 42, 43, 54, 55, 58], and 14 HCRU [12, 16, 19, 20, 28, 36, 37, 38, 43, 45, 47, 52, 63, 66].

#### 3.3.1 Direct Costs and HCRU

##### 3.3.1.1 Direct Costs

Five of nine studies (56%) found higher direct healthcare costs in PwO than those without [23, 24, 43, 54, 55]. The remaining four studies assessed PwO only [28, 42, 45, 68]. In a prospective study of US veterans, adjusted total costs per person per year (PPPY) were significantly higher in people with BMI ≥ 30 kg/m^2^ versus < 25 kg/m^2^ (2012–2015 USD: $45 683 [95% CI $44 087, $47 280] vs. $37 018 [$35 384, $38 651]; *p* < 0.01) [23]. In a retrospective cohort study of the National Health and Nutrition Examination Survey (NHANES) 2017–2018 linked to Medical Expenditure Panel Survey (MEPS), total healthcare costs PPPY (2019 USD) increased with obesity class—(average) class I: $6656; class II: $8169; class III: $9212—versus $5236 in individuals with healthy weight (*p* < 0.001) [43]. Among privately insured employees of major US industries, direct costs (2018 USD) PPPY were $8842 for those with class I obesity versus $7067 without obesity/overweight (difference, $1775; *p* < 0.05), and $18 548 for those with class III obesity (difference, $11 481; *p* < 0.05) [54]. Data suggest that direct healthcare costs are the primary contributors, largely driven by outpatient services and pharmacy expenditures [54, 55].

##### 3.3.1.2 Effects of Obesity Management Medications

Utilisation of obesity management medications (OMM) affected overall healthcare costs [28, 42, 68]. The MOVE! (Motivating Overweight/Obese Veterans Everywhere!) weight management program plus use of OMM was associated with lower mean total medical costs (including inpatient costs; excluding pharmacy costs) than MOVE! alone (2019 USD, PPPY: $18 182 vs. $20 075; *p* = 0.012) [28]. However, there were significantly higher annual pharmacy costs in the MOVE! plus OMM cohort ($4492 vs. $3274 in MOVE! alone), attributable to OMM use ($1302 vs. $10). Over 2 years, total healthcare costs (2019 USD, excluding OMM) decreased or remained stable among PwO using OMM, but increased in eligible non‐OMM users [68]. In PwO (class III), OMM users had a $891 *decrease* in total healthcare costs, versus a $1875 *increase* in non‐users (*p* = 0.012) [68], with similar trends when OMM costs were included (difference‐in‐differences in PwO [class III] OMM users vs. nonusers, −$5252 [95% CI −$7473, −$3031]). A cross‐sectional study found numerically higher total healthcare costs in OMM recipients versus eligible non‐recipients (2019 USD, PPPY: $8397 [95% CI: $4356, $12 438] vs. $6948 [$6545, $7351]; *p* = 0.475), mainly driven by higher outpatient ($2440 vs. $638; *p* = 0.272) and emergency room (ER) costs ($703 vs. $251; *p* = 0.252), but differences were not statistically significant [42].

##### 3.3.1.3 Effect of Weight‐Loss Interventions

Nonsurgical weight loss was associated with lower all‐cause healthcare costs after 1–2 years of weight loss, per a retrospective cohort study, driven mainly by inpatient/outpatient cost savings [24]; body weight loss of 10%–20% yielded average per‐person‐per‐month reductions of $263.01 (2018 USD) after 2 years [24]. Use of a digital weight‐management program (Noom Weight) was associated with lower per‐person healthcare costs after 24 months versus non‐users (2021 USD: $7367.97 vs. $8587.03; *p* = 0.005), excluding app fees [45].

##### 3.3.1.4 HCRU

HCRU was higher in PwO versus those without in five of the fourteen studies (36%) [16, 19, 43, 52, 66]. The remaining nine studies assessed PwO and overweight only [12, 20, 28, 36, 37, 38, 45, 47, 63]. This difference was most pronounced among individuals with higher obesity classes and was driven by increases in hospitalisation, length of stay (LOS) and intensive care use [12, 16, 19, 20, 37, 47]. Examples include a higher likelihood of obesity among patients hospitalised within 1 year of Veteran's Affairs enrollment [16], and a higher risk of readmission among Medicare beneficiaries with obesity aged ≥ 85 years (inpatient period 2011–2014) [19]. In a US database study, PwO with greater weight loss over 2 years reported fewer outpatient (< 7% weight loss, 37.8 [SD, 37.2] vs. > 15% weight loss, 35.0 [33.7]; *p* < 0.0001) and ED visits (< 7%, 0.58 [2.02] vs. > 15%, 0.56 [33.7]; *p* = 0.0003) per year [20]. A 5‐year weight‐management program in PwO reduced primary care, ambulatory clinic and overall healthcare interactions from baseline (*p* < 0.0001) [37].

#### 3.3.2 Indirect Costs

Despite variation in the type of indirect costs reported and methods used, all seven studies showed that obesity increased indirect cost burden, reflecting adverse impacts on employment and productivity. Five of the seven studies (71%) reported higher indirect costs in PwO versus those without [29, 43, 54, 55, 58]. The remaining two studies assessed PwO only [34, 42]. PwO experienced significantly longer spells of unemployment (mean: 16.3 weeks vs. 12.9 weeks in those without obesity) [29]. Employment rates were significantly lower in PwO (class III) versus people with healthy weight (59.9% vs. 64.9%; *p* = 0.002), and self‐reporting of inability to work (i.e., an incapacitating impairment preventing gainful employment) was greater with higher obesity classes (healthy, 10.3%; overweight, 11.2%; class I, 14.6%; class II, 17.8%; class III, 26.9%; *p* < 0.001) [43]. Across eight industries, PPPY costs for medical‐related absenteeism (calculated as worker's healthcare utilisation time × recorded wages) and disability (calculated from short‐ and long‐term disability claims) increased with obesity class (2018 USD, mean: reference group [proxy for healthy‐weight persons]: $1363 vs. class I, $2494; class II, $2475; class III, $3912) [55]. Higher classes of obesity had greater negative impacts on productivity (activity impairment measured by the Work Productivity and Impairment questionnaire: 21.0% for those with healthy weight vs. class I, 26.9%; class II, 31.8%; class III, 39.3%; *p* < 0.05) [58].

##### 3.3.2.1 Intervention Effects

Evidence for the effect of treatment interventions on indirect costs was limited. An intensive behavioural weight‐management program produced a non‐significant reduction in absenteeism after 6 months versus baseline (−2.56 h/month; *p* = 0.1) [34]. In addition to greater weight loss, OMM users among PwO were more likely to be employed than non‐users (75.6% vs. 68.7%; *p* = 0.339), although this difference was not statistically significant [42], but OMM users reported taking more sick days over 6 months (8.4 vs. 4.0 days in non‐users; *p* = 0.06) [42].

### 3.4 Studies Reporting Costs and HCRU in PwO Plus ORCs

#### 3.4.1 Direct Costs and HCRU

**FIGURE 2:**
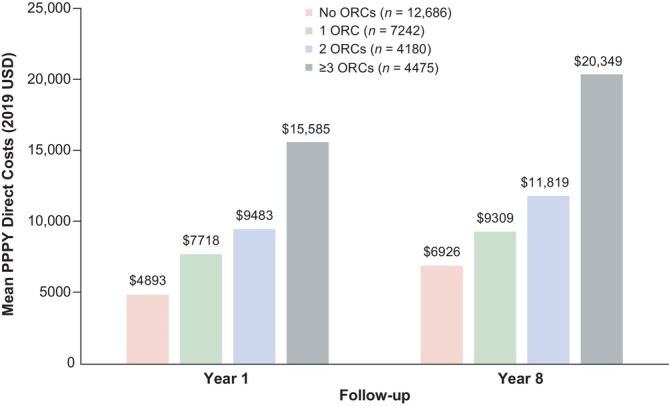
PPPY direct total healthcare costs in PwO by number of ORCs for Year 1 and Year 8 of follow‐up [49]. The analysis included adults with obesity identified between 2007 and 2012 who had ≥ 1 year of baseline data and ≥ 8 years of continuous post‐index follow‐up. ORC, obesity‐related complication; PPPY, per person per year; PwO, people living with obesity; USD, United States dollars.

Thirty‐eight studies reported general costs and HCRU in PwO with ORCs (Figure S1). PwO with multiple ORCs (*n* = 7 studies [25, 27, 48, 49, 64, 67, 69], Table 1) consistently incurred higher healthcare costs and greater HCRU than PwO without ORCs, with costs increasing as the number of ORCs increased. In a retrospective cohort study (2007–2012), PwO and three or more ORCs incurred over $13 000 (2019 USD) more in PPPY direct costs than PwO without ORCs, and costs increased over time (Figure 2) [49]. Costs were also higher in PwO and multiple ORCs with increasing obesity class [27], and cost differences between classes (adjusted for differences in Charlson Comorbidity Index score) widened over time (~27% higher for class III vs. class I at Year 1 increasing to ~41% by Year 8). Across obesity classes, outpatient services (including medical visits, laboratory tests, radiology examinations and ancillary services) accounted for approximately half the total healthcare costs.

Where data were available, PwO and specific ORCs generally incurred higher direct healthcare costs compared with those without obesity but with the same comorbid conditions. Total PPPY healthcare costs varied by ORC and data source (Table 2) [18, 25, 39, 49, 67]; however, the highest total healthcare costs were generally observed in PwO and CKD, heart failure with preserved ejection fraction (HFpEF), or T2D. Costs differed considerably by study, and the variation in methods used to assess total direct medical costs impeded between‐study comparisons.

**TABLE 2:** Reported total PPPY healthcare costs for PwO with various ORCs of interest.

Source	ORC	Total PPPY healthcare costs, USD	
		People living with obesity	People without obesity
Pearson‐Studdard et al. 2023. *International Journal of Obesity* ^a^ [49]	None	6926	—
	Chronic kidney disease	48 888	—
	Heart failure	27 535	—
	Type 2 diabetes	21 798	—
	Cardiovascular disease	20 380	—
	Osteoarthritis of the knee	18 083	—
Divino et al. 2021. *Journal of Managed Care & Specialty Pharmacy* ^b^ [25]	Heart failure with preserved ejection fraction	39 542	63 329
	Obstructive sleep apnea	12 866	13 709
	Dyslipidemia	12 674	9823
	Osteoarthritis of the knee	11 415	10 991
	Prediabetes	9681	12 440
	Type 2 diabetes	9832	14 759
	Polycystic ovarian syndrome	8717	5948
	Hypertension	6296	9480
	Musculoskeletal pain	6807	4942
Boye et al., 2020. *Journal of Diabetes and Its Complications* ^c^ [18]	Type 2 diabetes	26 624	24 838
Thorpe et al. 2021. *Journal of Occupational and Environmental Medicine* ^d^ [67]	Diabetes (type unspecified)	9127	—
	Arthritis	8681	—
	Back pain	7810	—
	Pulmonary disease	7331	—
	Hyperlipidemia	7054	—
	Hypertension	6736	—
Kumar and Encinosa, 2022. *Clinical Diabetes* ^e^ [39]	Without type 2 diabetes	5264	4453
	Type 2 diabetes	8599	7599

Abbreviations: BMI, body mass index; ORC, obesity‐related complication; PPPY, per‐person‐per‐year; PwO, people living with obesity; USD, United States dollars.

^a^ Measured in 2019 USD at Year 8 in people with BMI ≥ 30 and < 70 kg/m^2^.

^b^ Measured in 2017 USD at baseline in people with BMI ≥ 40 kg/m^2^ (with obesity) and people with BMI 18.5–24.9 kg/m^2^ (without obesity).

^c^ Measured in 2018 USD in people with BMI ≥ 30 kg/m^2^ (with obesity) versus BMI 18.5–30 kg/m^2^ (without obesity).

^d^ Measured 2019 USD in people with BMI ≥ 30 kg/m^2^.

^e^ Measured in people with BMI ≥ 35 kg/m^2^ (with obesity) versus people with BMI 20 to < 25 kg/m^2^ (without obesity).

Weight loss among PwO and ORCs was associated with reductions in annual medical expenditures. The largest annual savings in total healthcare expenditure were reported for diabetes and hypertension (2019 USD: $752 and $367 per 1 kg/m^2^ lost, respectively) [67]. Cost savings increased with greater weight loss and higher baseline BMI among individuals with diabetes [67]. For all conditions, greater weight loss was associated with greater reduction in total healthcare costs; for example, for PwO and diabetes with a baseline BMI of 40 kg/m^2^, a 5% BMI reduction was associated with annual cost savings of $2665 versus savings of $8443 with a 20% BMI reduction [67].

##### 3.4.1.1 Hospitalisation Costs and LOS

**FIGURE 3:**
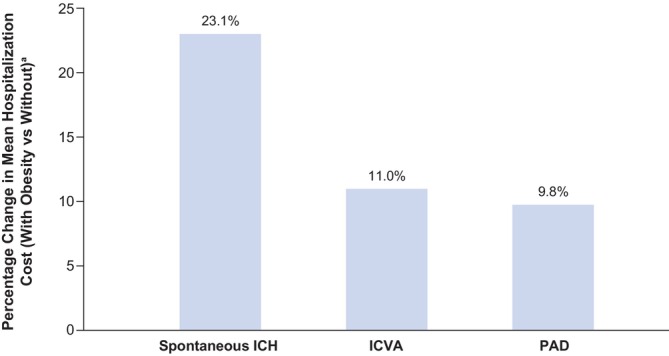
Percentage increase in PPPY hospitalisation charges in PwO with ORCs versus people without obesity (or with a lower BMI^a^) with the same conditions [32, 41, 60]. ^a^PAD study compares people with BMI > 25 kg/m^2^ versus < 25 kg/m^2^. Spontaneous ICH: *N* = 123 415; comprised of 117 923 people with healthy weight, 3687 people with obesity (BMI 30 to 39.9 kg/m^2^), and 1805 people with severe obesity (BMI ≥ 40 kg/m^2^). ICVA: *N* = 1 032 240; comprised of 895 260 people without obesity (BMI < 30 kg/m^2^), and 136 980 people with obesity (BMI ≥ 30 kg/m^2^). PAD: *N* = 248 228; comprised of 206 610 people with BMI < 25 kg/m^2^, and 41 618 people with obesity or BMI > 25 kg/m^2^. BMI, body mass index; ICH, intracerebral haemorrhage; ICVA, ischemic cerebrovascular accident; ORC, obesity‐related complication; PAD, peripheral arterial disease; PPPY, per person per year; PwO, people living with obesity.

PwO and ORCs generally incurred higher hospitalisation costs (Figure 3 [32, 41, 60]) and longer LOS (Figure S2) than people with the same conditions but without obesity. Three studies reported higher mean PPPY hospitalisation costs in PwO and ORCs of interest [32, 41, 60], with the largest relative increase observed for spontaneous intracerebral haemorrhage, followed by ischemic cerebrovascular accident and peripheral arterial disease (PAD) [32, 41, 60]. PwO and ORCs experienced longer mean hospital LOS than those without obesity [21, 30, 41, 57, 60]. The longest LOS was reported among PwO with venous thromboembolism, with a mean LOS of 25 days for BMI ≥ 40 kg/m^2^ versus 20 days for BMI 30–34.9 kg/m^2^ [27].

#### 3.4.2 ORCs of Interest

##### 3.4.2.1 Diabetes‐Related Complications

Seven studies evaluated higher direct healthcare costs among PwO and diabetes‐related complications, all of which reported higher costs for PwO than for persons without obesity but with similar complications [18, 30, 33, 39, 53, 58, 71]. Increased HCRU in PwO and T2D was driven by more frequent hospitalisations, longer LOS and increased outpatient care [18, 30, 33, 53]. In a large claims‐based analysis of adults with T2D, PwO had significantly higher annual diabetes‐related total costs (2018 USD: $8987 vs. $8057; *p* < 0.0001), and all‐cause total costs ($26 624 vs. $24 838; *p* < 0.0001) than adults with healthy weight or overweight [18]. PwO also used more medical resources than the cohort with healthy weight/overweight, with significantly more diabetes‐related and all‐cause hospitalisations, ER and office visits and longer hospital LOS [18]. PwO (class III) and diabetes‐related foot complications had a longer LOS (adjusted mean difference [aMD]: 0.47 [95% CI 0.13, 0.81]) and higher hospital costs (aMD [2014 USD]: $3205 [$373, $6037]) than those without class III obesity [30]. In addition, PwO (class II/III) and T2D reported greater work and activity impairment than those without T2D (43.2% vs. 33.0%, respectively), contributing to a higher indirect cost burden [58].

##### 3.4.2.2 CV Complications

Thirteen studies evaluated costs and HCRU in PwO with CV complications [13, 14, 15, 21, 31, 40, 41, 50, 51, 59, 61, 62, 65]. Direct costs varied by medical condition and treatment [13, 14, 40, 51]. Overall, PwO experienced greater HCRU than those without obesity [31, 41, 59]. For example, patients with PAD and a BMI > 25 kg/m^2^ had a longer mean hospital LOS than those with a BMI < 25 kg/m^2^ (6.5 vs. 5.8 days; *p* < 0.0001) and higher total hospital charges (2016 USD: $73 046 vs. $66 541; *p* < 0.0001) [41]. However, findings varied by condition and setting; for example, obesity was not associated with higher readmission rates following acute myocardial infarction [59], or in patients with cardiogenic shock requiring mechanical circulatory support (MCS) [62]. Patients without obesity were ~21% less likely to be readmitted to hospital than PwO (OR 0.788; 95% CI 0.751, 0.827; *p* < 0.0001), and were more likely to be readmitted regardless of initial LOS [59]. In patients with cardiogenic shock requiring MCS, obesity of any class had no independent association with 30‐day readmission rates [62].

##### 3.4.2.3 Stroke Complications

Five studies reported outcomes in PwO and stroke‐related complications (Table 1) [32, 44, 57, 60, 70]. Two studies reported higher direct costs in PwO versus those without obesity [32, 60]. Trends in mean hospital LOS for stroke complications were varied, and several studies reported no difference by obesity status [32, 44, 70]. However, one study reported shorter LOS among PwO and stroke consistent with a reverse J‐shaped curve (mean LOS 7.45 days for BMI 20–25 kg/m^2^, 5.65 for 26–30 kg/m^2^, 4.90 for 31–35 kg/m^2^ and 5.59 for > 40 kg/m^2^; *p* < 0.001) [57]. These findings may be explained by the ‘obesity paradox’, which postulates potential cardioprotective effects of obesity in populations with certain chronic conditions, such as stroke [57, 72, 73]. Despite variable LOS findings, hospitalised PwO with stroke complications generally incurred higher hospital charges than those without obesity (*p* < 0.001) [32].

##### 3.4.2.4 Other ORCs

Additional ORCs evaluated included OA [35], liver disease (MASLD) [46] and pancreas/gallbladder disease [17, 22]. Costs and HCRU were substantial in PwO, and increased with obesity severity. Among PwO with OA increasing obesity severity was associated with increased total healthcare expenditures (adjusted mean 2015 USD: obesity class I, $19 135; class II, $20 679; class III, $23 372) and higher all‐cause hospitalisation rates including increased orthopaedic surgery–related admissions (obesity class I, 26.4%; class II, 28.9%; class III, 32.0%) [35]. Among individuals with non‐alcoholic fatty liver disease (MASLD), PwO incurred higher direct healthcare costs than those without obesity (mean healthcare cost at baseline, 2010–2014 USD: with obesity, $13 221.1 [SD 139.9] vs. without obesity, $12 980.8 [SD 151.1]; *p* < 0.0001) [46]. However, mean hospital LOS was 4.4 days in PwO and 5.1 days in persons without obesity (*p* < 0.0001), and the difference reached statistically significance due to the large sample size (*N* ~ 195 000 hospitalisations) [46]. In PwO with acute pancreatitis, direct medical costs were substantial and varied by obesity severity, while findings on hospital LOS were mixed [17, 22]. One study reported longer LOS for PwO (class III) versus those without (5.41 vs. 4.68 days; *p* < 0.001) [17]; another study reported shorter LOS in PwO (all obesity classes) versus people without obesity (5.8 vs. 8.2 days; *p* < 0.0001) [22].

## 4 Discussion

The findings of this SLR suggest that obesity and associated ORCs impose a substantial economic burden on the US healthcare system. Both direct healthcare costs and HCRU increase with higher obesity class and a greater number of ORCs. Direct costs were driven primarily by higher inpatient/outpatient expenses, while HCRU was driven by higher hospitalisation rates, longer LOS, greater intensive care use and increased ongoing medical/outpatient care in PwO compared with those without overweight or obesity. These findings underscore the need for comprehensive obesity management strategies that integrate evidence‐based pharmacological and surgical treatments with structured lifestyle interventions to improve outcomes and potentially mitigate healthcare costs. While obesity and its complications are recognised as chronic conditions, this review did not assess disease‐specific treatment or management strategies, focusing instead on associated economic and healthcare resource implications.

Expanded access to effective obesity treatments may reduce the economic burden of obesity and ORCs. Despite growing evidence for their clinical effectiveness, OMM are often not covered by many commercial insurance plans, Medicare, or Medicaid [74]. Improved insurance coverage and reduced out‐of‐pocket costs for OMM have been associated with better treatment adherence and greater weight loss [75, 76]. Additionally, bariatric surgery [77, 78, 79] and OMM [68, 80] have been linked to healthcare cost savings in several studies. Alternative care delivery and reimbursement models including shared savings programs and population health‐based approaches may further reduce the cost burden of obesity care.

Several obesity pharmacotherapies also confer benefits across multiple ORCs. In particular, GLP‐1 receptor agonist–based OMM have demonstrated benefits in T2D, cardiorenal disease, cardiovascular events, HFpEF, stroke, metabolic liver disease and sleep apnea, with ongoing investigation in PAD and neurodegenerative conditions [81]. Further research is needed to determine whether concomitant treatment of obesity and ORCs with these agents translates into additional cost savings.

Despite their benefits, pharmaceutical and surgical treatments for obesity remain underutilised. Historically, uptake of OMM among eligible individuals has been minimal (~1%) [42, 82]; although, utilisation has increased in recent years, accompanied by supply shortages that have constrained access [83, 84]. Bariatric surgery utilisation has remained low among eligible patients and has declined in more recent years [85, 86]. These patterns highlight persistent barriers to effective obesity care including limited clinician training, gaps in clinical expertise and inadequate insurance coverage.

This SLR has several limitations. Included studies were highly heterogeneous in design, populations, interventions, timeframes and data sources for HCRU, costs and productivity, precluding quantitative synthesis and limiting conclusions to overall trends. Selection and publication bias cannot be excluded, given the focus on English‐language, US‐based studies indexed in Embase or MEDLINE, and the exclusion of grey and unpublished literature. These factors may have limited the comprehensiveness of this review. The potential influence of demographic factors, insurance coverage and the COVID‐19 pandemic on the economic burden and HCRU in PwO versus persons without obesity was not assessed. Furthermore, while obesity and its complications are recognised as chronic conditions, this review did not assess disease‐specific treatment and management strategies, focusing instead on associated economic and healthcare resource implications.

## 5 Conclusions

Obesity, overweight, and their associated ORCs are linked to a substantial and increasing economic burden in the US, driven by elevated direct and indirect healthcare costs and HCRU. Expanding access to effective, evidence‐based obesity treatments is critical to reducing the strain on the healthcare system.

## Author Contributions

All authors were involved in the conception of the work and critically reviewed and edited the final report of the systematic literature review. All authors reviewed the manuscript at all stages of development and provided final approval of the version submitted for publication. All authors agree to be accountable for all aspects of the work.

## Funding

The SLR and writing support for this manuscript were contracted and funded by Boehringer Ingelheim Pharmaceuticals Inc. (BIPI).

## Ethics Statement

The protocol was prospectively registered with the International Prospective Register of Systematic Reviews (PROSPERO; registration number: CRD42024491292).

## Conflicts of Interest

J.P.A. has served as a consultant/advisor for AbbVie, Boehringer Ingelheim, Eli Lilly, Nestlé, Novo Nordisk and Wave Life Sciences. J.D.A. serves as a consultant for Amgen, Boehringer Ingelheim, Eli Lilly, Nestlé Healthcare Nutrition, Novo Nordisk and WeightWatchers, and has received research support from Amgen, Boehringer Ingelheim, Eli Lilly, Epitomee, KVKTech, Nestlé Healthcare Nutrition, Novo Nordisk and WeightWatchers. C.G. and S.E.‐H. have nothing to disclose.

## Supporting information


**Data S1:** dom70602‐sup‐0001‐Supinfo.docx.

**Figure S1:** dom70602‐sup‐0002‐Figure S1.pdf.

**Figure S2:** dom70602‐sup‐0003‐Figure S2.pdf.

## Data Availability

All relevant data are in the public domain and shared within the manuscript.
