# Supplementary material for: Healthcare Resource Utilisation and Cost of Obesity and Related Complications in the United States: A Systematic Literature Review
Source: Diabetes Obes Metab. 2026 Mar 9;28(5):4121–36. doi: 10.1111/dom.70602 (PMC13071262; doi:10.1111/dom.70602)
Supplement: Supplementary file 1 — Data S1: dom70602‐sup‐0001‐Supinfo.docx. [file DOM-28-4121-s003.docx]

SUPPLEMENTARY MATERIAL

Healthcare resource utilization and cost of obesity and related complications in the United States: a systematic literature review

Jaime P. Almandoz^1,^^[[1]](#footnote-1)^*, Jamy D. Ard^2^, Chellse Gazda^1^, Shenelle Edwards-Hampton^2^

^1^ University of Texas Southwestern Medical Center, Dallas, TX, USA
^2^ Wake Forest School of Medicine, Winston Salem, NC, USA

**CONTENTS**

[TABLES 2](#_Toc212446849)

[**TABLE S1** Embase^®^ search strategy. 2](#_Toc212446850)

[**TABLE S2** Medline^®^ search strategy. 4](#_Toc212446851)

[**TABLE S3** Evidence-Based Medicine Reviews search strategy (Cochrane Central Register of Controlled Trials). 6](#_Toc212446852)

[**TABLE S4** Evidence-Based Medicine Reviews search strategy (Cochrane Database of Systematic Reviews). 8](#_Toc212446853)

[**TABLE S5** Quality appraisal of cost and HCRU studies using Molinier et al.^1^ 10](#_Toc212446854)

[FIGURES 13](#_Toc212446855)

[**FIGURE S1** Number of studies included in SLR, by ORCs. 13](#_Toc212446856)

[**FIGURE S2** Hospital LOS in PwO with ORCs,^11,20,31,47,50^ 14](#_Toc212446857)

[REFERENCES 15](#_Toc212446858)

# TABLES

## **TABLE S1** Embase^®^ search strategy.

| # | Query | Results from  **Jan 26, 2024** |
| --- | --- | --- |
| **1** | exp *obesity/ | 300,403 |
| **2** | *weight reduction/ | 26,455 |
| **3** | (obesity or obese).ti. | 209,982 |
| **4** | (weight adj2 (los* or reduc*)).ti,ab. | 228,109 |
| **5** | (overweight or over-weight or over weight or overeating or over eating or over-eating).ti. | 30,148 |
| **6** | or/1-5 | 515,182 |
| **7** | Cost of illness/ | 21,581 |
| **8** | Health care cost/ | 231,581 |
| **9** | Health care financing/ | 14,048 |
| **10** | Hospital cost/ | 25,911 |
| **11** | (cost adj estimate$).ti,ab. | 4,254 |
| **12** | ((cost adj variable$) or (economi$ adj impact$)).ti,ab. | 22,774 |
| **13** | (unit adj cost$).ti,ab. | 5,632 |
| **14** | ((indirect or direct or employe*) adj (cost* or resource* or benefit*)).ti,ab. | 26,370 |
| **15** | employment status/ | 22,976 |
| **16** | hospitalization/ | 550,401 |
| **17** | length of stay/ | 281,032 |
| **18** | health care utilization/ | 97,969 |
| **19** | ("resource utilization" or "resource utilisation" or "resource use" or "healthcare utilisation" or "healthcare utilization" or "health care utilisation" or "health care utilization" or "health care use" or "healthcare use" or "health service use" or "health services use").ti,ab. | 79,099 |
| **20** | (((days or work or week or weeks or month or months or year or years) adj (loss or lost)) or employment or unemployment or "sick leave" or hospitaliz$ or hospitalis$).ti,ab. | 696,433 |
| **21** | ((absent or sick) adj (day or days or week or weeks or month or months or year or years or period or periods)).ti,ab. | 1,173 |
| **22** | ((loss or lost or losing or reduc*) adj (work or career or income)).ti,ab. | 4,285 |
| **23** | (impact adj (work or working or career$1 or income)).ti,ab. | 329 |
| **24** | (((hospital$1 or inpatient or "in patient" or outpatient or "out patient" or emergency or "intensive care" or ICU or ITU) adj (visit or visits or admit* or admission$1 or readmission$1 or readmit* or appointment$1 or schedule$1)) or (length adj2 stay)).ti,ab. | 315,175 |
| **25** | Productivity/ | 50,974 |
| **26** | Absenteeism/ or absentee*.ti,ab. | 23,621 |
| **27** | presenteeism/ or presentee*.ti,ab. | 3,858 |
| **28** | time off.ti,ab. | 3,785 |
| **29** | days off.ti,ab. | 3,002 |
| **30** | or/7-29 | 1,646,176 |
| **31** | (conference abstract or editorial or comment* or letter or review or note or case study or case studies or case reports).pt. or (case stud* or case report* or congress or symposium).ti,ab,sh. | 13,734,915 |
| **32** | ((animal$ not human$) or nonhuman or human cell).sh,hw. or exp preclinical study/ | 10,779,140 |
| **33** | (in vitro or rat or rats or rodent or rodents or mouse or mice or murine or dog or dogs or canine or pig or pigs or porcine).ti,ab. | 5,349,357 |
| **34** | 32 or 33 | 11,946,406 |
| **35** | 31 or 34 | 23,018,573 |
| **36** | afghanistan/ or exp africa/ or argentina/ or bahrain/ or bangladesh/ or bhutan/ or bolivia/ or borneo/ or exp brazil/ or brunei darussalam/ or exp china/ or colombia/ or ecuador/ or equatorial guinea/ or guinea/ or guinea-bissau/ or guyana/ or exp india/ or exp indonesia/ or iran/ or exp iraq/ or jordan/ or kazakhstan/ or kuwait/ or kyrgyzstan/ or laos/ or lebanon/ or exp malaysia/ or mongolia/ or myanmar/ or nepal/ or north korea/ or oman/ or exp pakistan/ or papua new guinea/ or paraguay/ or peru/ or qatar/ or philippines.mp. or exp russian federation/ or saudi arabia/ or singapore/ or sri lanka/ or suriname/ or syrian arab republic/ or taiwan/ or tajikistan/ or thailand/ or timor-leste/ or turkmenistan/ or exp united arab emirates/ or uruguay/ or exp uzbekistan/ or venezuela/ or viet nam/ or yemen/ or exp "australia and new zealand"/ or austria/ or exp "baltic states"/ or exp belgium/ or chile/ or "czech republic"/ or denmark/ or europe/ or exp finland/ or exp france/ or exp germany/ or greece/ or hungary/ or iceland/ or ireland/ or israel/ or exp italy/ or japan/ or korea/ or luxembourg/ or netherlands/ or exp norway/ or exp portugal/ or scandinavia/ or slovakia/ or slovenia/ or "south korea"/ or exp spain/ or sweden/ or switzerland/ or exp "turkey (republic)"/ or exp united kingdom/ or western europe/ [mp=title, abstract, heading word, drug trade name, original title, device manufacturer, drug manufacturer, device trade name, keyword heading word, floating subheading word, candidate term word] | 3,768,574 |
| **37** | european union/ | 31,794 |
| **38** | 36 or 37 | 3,786,766 |
| **39** | 6 and 30 | 27,316 |
| **40** | 39 not 35 | 11,921 |
| **41** | limit 40 to yr="2019 -Current" | 4,307 |
| **42** | 41 not 38 | 3,625 |

## **TABLE S2** Medline^®^ search strategy.

| # | Query | Results from Jan 26, 2024 |
| --- | --- | --- |
| **1** | *Obesity/ | 138,392 |
| **2** | exp *overweight/ | 191,595 |
| **3** | *weight loss/ | 17,454 |
| **4** | exp *weight reduction programs/ | 2,379 |
| **5** | (obesity or obese).ti. | 152,305 |
| **6** | (weight adj2 (los* or reduc*)).ti,ab. | 143,091 |
| **7** | (overweight or over-weight or over weight or overeating or over eating or over-eating).ti. | 22,055 |
| **8** | or/1-7 | 337,119 |
| **9** | "Cost of Illness"/ | 31,951 |
| **10** | Health Care Costs/ | 44,665 |
| **11** | Hospital Costs/ | 12,010 |
| **12** | (cost adj estimate$).ti,ab. | 2,815 |
| **13** | ((cost adj variable$) or (economi$ adj impact$)).ti,ab. | 15,982 |
| **14** | (unit adj cost$).ti,ab. | 3,193 |
| **15** | ((indirect or direct or employe*) adj (cost* or resource* or benefit*)).ti,ab. | 16,479 |
| **16** | Employment/ | 51,098 |
| **17** | Hospitalization/ | 138,264 |
| **18** | "Length of Stay"/ | 103,736 |
| **19** | ("resource utilization" or "resource utilisation" or "resource use" or "healthcare utilisation" or "healthcare utilization" or "health care utilisation" or "health care utilization" or "health care use" or "healthcare use" or "health service use" or "health services use").ti,ab. | 50,487 |
| **20** | (((days or work or week or weeks or month or months or year or years) adj (loss or lost)) or employment or unemployment or "sick leave" or hospitaliz$ or hospitalis$).ti,ab. | 440,683 |
| **21** | ((absent or sick) adj (day or days or week or weeks or month or months or year or years or period or periods)).ti,ab. | 725 |
| **22** | ((loss or lost or losing or reduc*) adj (work or career or income)).ti,ab. | 3,124 |
| **23** | (impact adj (work or working or career$1 or income)).ti,ab. | 232 |
| **24** | (((hospital$1 or inpatient or "in patient" or outpatient or "out patient" or emergency or "intensive care" or ICU or ITU) adj (visit or visits or admit* or admission$1 or readmission$1 or readmit* or appointment$1 or schedule$1)) or (length adj2 stay)).ti,ab. | 175,490 |
| **25** | Efficiency/ | 15,758 |
| **26** | Absenteeism/ or absentee*.ti,ab. | 14,197 |
| **27** | presenteeism/ or presentee*.ti,ab. | 2,425 |
| **28** | time off.ti,ab. | 2,454 |
| **29** | days off.ti,ab. | 1,513 |
| **30** | or/9-29 | 848,261 |
| **31** | (conference abstract or editorial or comment* or letter or review or note or case study or case studies or case reports).pt. or (case stud* or case report* or congress or symposium).ti,ab,sh. | 7,746,364 |
| **32** | ((animal$ not human$) or nonhuman or human cell).sh,hw. or preclinical study.mp. | 5,147,871 |
| **33** | (in vitro or rat or rats or rodent or rodents or mouse or mice or murine or dog or dogs or canine or pig or pigs or porcine).ti,ab. | 4,473,823 |
| **34** | 32 or 33 | 6,997,802 |
| **35** | 31 or 34 | 14,243,089 |
| **36** | afghanistan/ or exp africa/ or albania/ or andorra/ or antarctic regions/ or argentina/ or exp asia, central/ or exp asia, northern/ or exp asia, southeastern/ or exp atlantic islands/ or bahrain/ or bangladesh/ or bhutan/ or bolivia/ or borneo/ or "bosnia and herzegovina"/ or brazil/ or bulgaria/ or exp central america/ or exp china/ or colombia/ or "commonwealth of independent states"/ or croatia/ or "democratic people's republic of korea"/ or ecuador/ or gibraltar/ or guyana/ or exp india/ or indonesia/ or iran/ or iraq/ or jordan/ or kosovo/ or kuwait/ or lebanon/ or liechtenstein/ or macau/ or "macedonia (republic)"/ or exp melanesia/ or moldova/ or monaco/ or mongolia/ or montenegro/ or nepal/ or netherlands antilles/ or new guinea/ or oman/ or pakistan/ or paraguay/ or peru/ or philippines/ or qatar/ or "republic of belarus"/ or romania/ or exp russia/ or saudi arabia/ or serbia/ or sri lanka/ or suriname/ or syria/ or taiwan/ or exp transcaucasia/ or ukraine/ or uruguay/ or united arab emirates/ or exp ussr/ or venezuela/ or yemen/ or australasia/ or exp australia/ or austria/ or exp baltic states/ or belgium/ or exp canada/ or chile/ or czech republic/ or europe/ or exp france/ or exp germany/ or greece/ or hungary/ or ireland/ or israel/ or exp italy/ or exp japan/ or korea/ or luxembourg/ or mexico/ or netherlands/ or new zealand/ or poland/ or portugal/ or exp "republic of korea"/ or exp "scandinavian and nordic countries"/ or slovakia/ or slovenia/ or spain/ or switzerland/ or turkey/ or exp united kingdom/ | 3,424,045 |
| **37** | european union/ | 17,885 |
| **38** | 36 or 37 | 3,433,557 |
| **39** | 8 and 30 | 11,942 |
| **40** | 39 not 35 | 9,705 |
| **41** | limit 40 to yr="2019 -Current" | 3,371 |
| **42** | 41 not 38 | 2,749 |

## **TABLE S3** Evidence-Based Medicine Reviews search strategy (Cochrane Central Register of Controlled Trials).

| # | Query | Results from Jan 26, 2024 |
| --- | --- | --- |
| 1 | *Obesity/ | 0 |
| 2 | exp *overweight/ | 12,384 |
| 3 | *weight loss/ | 0 |
| 4 | exp *weight reduction programs/ | 0 |
| 5 | (obesity or obese).ti. | 21,609 |
| 6 | (weight adj2 (los* or reduc*)).ti,ab. | 27,048 |
| 7 | (overweight or over-weight or over weight or overeating or over eating or over-eating).ti. | 7,891 |
| 8 | or/1-7 | 46,454 |
| 9 | "Cost of Illness"/ | 1,074 |
| 10 | Health Care Costs/ | 2,812 |
| 11 | Hospital Costs/ | 712 |
| 12 | (cost adj estimate$).ti,ab. | 270 |
| 13 | ((cost adj variable$) or (economi$ adj impact$)).ti,ab. | 1,282 |
| 14 | (unit adj cost$).ti,ab. | 768 |
| 15 | ((indirect or direct or employe*) adj (cost* or resource* or benefit*)).ti,ab. | 2,481 |
| 16 | Employment/ | 1,005 |
| 17 | Hospitalization/ | 10,561 |
| 18 | "Length of Stay"/ | 9,876 |
| 19 | ("resource utilization" or "resource utilisation" or "resource use" or "healthcare utilisation" or "healthcare utilization" or "health care utilisation" or "health care utilization" or "health care use" or "healthcare use" or "health service use" or "health services use").ti,ab. | 9,397 |
| 20 | (((days or work or week or weeks or month or months or year or years) adj (loss or lost)) or employment or unemployment or "sick leave" or hospitaliz$ or hospitalis$).ti,ab. | 65,438 |
| 21 | ((absent or sick) adj (day or days or week or weeks or month or months or year or years or period or periods)).ti,ab. | 217 |
| 22 | ((loss or lost or losing or reduc*) adj (work or career or income)).ti,ab. | 465 |
| 23 | (impact adj (work or working or career$1 or income)).ti,ab. | 28 |
| 24 | (((hospital$1 or inpatient or "in patient" or outpatient or "out patient" or emergency or "intensive care" or ICU or ITU) adj (visit or visits or admit* or admission$1 or readmission$1 or readmit* or appointment$1 or schedule$1)) or (length adj2 stay)).ti,ab. | 27,628 |
| 25 | Efficiency/ | 407 |
| 26 | Absenteeism/ or absentee*.ti,ab. | 1,791 |
| 27 | presenteeism/ or presentee*.ti,ab. | 544 |
| 28 | time off.ti,ab. | 482 |
| 29 | days off.ti,ab. | 679 |
| 30 | or/9-29 | 109,894 |
| 31 | (conference abstract or editorial or comment* or letter or review or note or case study or case studies or case reports).pt. or (case stud* or case report* or congress or symposium).ti,ab,sh. | 48,156 |
| 32 | ((animal$ not human$) or nonhuman or human cell).sh,hw. or preclinical study.mp. | 58,398 |
| 33 | (in vitro or rat or rats or rodent or rodents or mouse or mice or murine or dog or dogs or canine or pig or pigs or porcine).ti,ab. | 36,825 |
| 34 | 32 or 33 | 89,235 |
| 35 | 31 or 34 | 135,965 |
| 36 | afghanistan/ or exp africa/ or albania/ or andorra/ or antarctic regions/ or argentina/ or exp asia, central/ or exp asia, northern/ or exp asia, southeastern/ or exp atlantic islands/ or bahrain/ or bangladesh/ or bhutan/ or bolivia/ or borneo/ or "bosnia and herzegovina"/ or brazil/ or bulgaria/ or exp central america/ or exp china/ or colombia/ or "commonwealth of independent states"/ or croatia/ or "democratic people's republic of korea"/ or ecuador/ or gibraltar/ or guyana/ or exp india/ or indonesia/ or iran/ or iraq/ or jordan/ or kosovo/ or kuwait/ or lebanon/ or liechtenstein/ or macau/ or "macedonia (republic)"/ or exp melanesia/ or moldova/ or monaco/ or mongolia/ or montenegro/ or nepal/ or netherlands antilles/ or new guinea/ or oman/ or pakistan/ or paraguay/ or peru/ or philippines/ or qatar/ or "republic of belarus"/ or romania/ or exp russia/ or saudi arabia/ or serbia/ or sri lanka/ or suriname/ or syria/ or taiwan/ or exp transcaucasia/ or ukraine/ or uruguay/ or united arab emirates/ or exp ussr/ or venezuela/ or yemen/ or australasia/ or exp australia/ or austria/ or exp baltic states/ or belgium/ or exp canada/ or chile/ or czech republic/ or europe/ or exp france/ or exp germany/ or greece/ or hungary/ or ireland/ or israel/ or exp italy/ or exp japan/ or korea/ or luxembourg/ or mexico/ or netherlands/ or new zealand/ or poland/ or portugal/ or exp "republic of korea"/ or exp "scandinavian and nordic countries"/ or slovakia/ or slovenia/ or spain/ or switzerland/ or turkey/ or exp united kingdom/ | 100,902 |
| 37 | european union/ | 191 |
| 38 | 36 or 37 | 101,028 |
| 39 | 8 and 30 | 1,592 |
| 40 | 39 not 35 | 1,540 |
| 41 | limit 40 to yr="2019 -Current" | 505 |
| 42 | 41 not 38 | 476 |

## **TABLE S4** Evidence-Based Medicine Reviews search strategy (Cochrane Database of Systematic Reviews).

| # | Query | Results from Jan 26, 2024 |
| --- | --- | --- |
| 1 | [exp *obesity/] | 0 |
| 2 | [*weight reduction/] | 0 |
| 3 | (obesity or obese).ti. | 77 |
| 4 | (weight adj2 (los* or reduc*)).ti,ab. | 130 |
| 5 | (overweight or over-weight or over weight or overeating or over eating or over-eating).ti. | 44 |
| 6 | or/1-5 | 182 |
| 7 | [Cost of illness/] | 0 |
| 8 | [Health care cost/] | 0 |
| 9 | [Health care financing/] | 0 |
| 10 | [Hospital cost/] | 0 |
| 11 | (cost adj estimate$).ti,ab. | 3 |
| 12 | ((cost adj variable$) or (economi$ adj impact$)).ti,ab. | 13 |
| 13 | (unit adj cost$).ti,ab. | 2 |
| 14 | ((indirect or direct or employe*) adj (cost* or resource* or benefit*)).ti,ab. | 17 |
| 15 | [employment status/] | 0 |
| 16 | [hospitalization/] | 0 |
| 17 | [length of stay/] | 0 |
| 18 | [health care utilization/] | 0 |
| 19 | ("resource utilization" or "resource utilisation" or "resource use" or "healthcare utilisation" or "healthcare utilization" or "health care utilisation" or "health care utilization" or "health care use" or "healthcare use" or "health service use" or "health services use").ti,ab. | 151 |
| 20 | (((days or work or week or weeks or month or months or year or years) adj (loss or lost)) or employment or unemployment or "sick leave" or hospitaliz$ or hospitalis$).ti,ab. | 618 |
| 21 | ((absent or sick) adj (day or days or week or weeks or month or months or year or years or period or periods)).ti,ab. | 1 |
| 22 | ((loss or lost or losing or reduc*) adj (work or career or income)).ti,ab. | 7 |
| 23 | (impact adj (work or working or career$1 or income)).ti,ab. | 0 |
| 24 | (((hospital$1 or inpatient or "in patient" or outpatient or "out patient" or emergency or "intensive care" or ICU or ITU) adj (visit or visits or admit* or admission$1 or readmission$1 or readmit* or appointment$1 or schedule$1)) or (length adj2 stay)).ti,ab. | 457 |
| 25 | [Productivity/] | 0 |
| 26 | [Absenteeism/ or absentee*.ti,ab.] | 0 |
| 27 | [presenteeism/ or presentee*.ti,ab.] | 0 |
| 28 | time off.ti,ab. | 12 |
| 29 | days off.ti,ab. | 11 |
| 30 | or/7-29 | 1,130 |
| 31 | [(conference abstract or editorial or comment* or letter or review or note or case study or case studies or case reports).pt. or (case stud* or case report* or congress or symposium).ti,ab,sh.] | 0 |
| 32 | [((animal$ not human$) or nonhuman or human cell).sh,hw. or exp preclinical study/] | 0 |
| 33 | (in vitro or rat or rats or rodent or rodents or mouse or mice or murine or dog or dogs or canine or pig or pigs or porcine).ti,ab. | 127 |
| 34 | 32 or 33 | 127 |
| 35 | 31 or 34 | 127 |
| 36 | [afghanistan/ or exp africa/ or argentina/ or bahrain/ or bangladesh/ or bhutan/ or bolivia/ or borneo/ or exp brazil/ or brunei darussalam/ or exp china/ or colombia/ or ecuador/ or equatorial guinea/ or guinea/ or guinea-bissau/ or guyana/ or exp india/ or exp indonesia/ or iran/ or exp iraq/ or jordan/ or kazakhstan/ or kuwait/ or kyrgyzstan/ or laos/ or lebanon/ or exp malaysia/ or mongolia/ or myanmar/ or nepal/ or north korea/ or oman/ or exp pakistan/ or papua new guinea/ or paraguay/ or peru/ or qatar/ or philippines.mp. or exp russian federation/ or saudi arabia/ or singapore/ or sri lanka/ or suriname/ or syrian arab republic/ or taiwan/ or tajikistan/ or thailand/ or timor-leste/ or turkmenistan/ or exp united arab emirates/ or uruguay/ or exp uzbekistan/ or venezuela/ or viet nam/ or yemen/ or exp "australia and new zealand"/ or austria/ or exp "baltic states"/ or exp belgium/ or chile/ or "czech republic"/ or denmark/ or europe/ or exp finland/ or exp france/ or exp germany/ or greece/ or hungary/ or iceland/ or ireland/ or israel/ or exp italy/ or japan/ or korea/ or luxembourg/ or netherlands/ or exp norway/ or exp portugal/ or scandinavia/ or slovakia/ or slovenia/ or "south korea"/ or exp spain/ or sweden/ or switzerland/ or exp "turkey (republic)"/ or exp united kingdom/ or western europe/ [mp=title, abstract, heading word, drug trade name, original title, device manufacturer, drug manufacturer, device trade name, keyword heading word, floating subheading word, candidate term word]] | 0 |
| 37 | [european union/] | 0 |
| 38 | 36 or 37 | 0 |
| 39 | 6 and 30 | 12 |
| 40 | 39 not 35 | 12 |

## **TABLE S5** Quality appraisal of cost and HCRU studies using Molinier et al.^1^

|  | **1)**  Was a clear definition of the illness given? | **2)**  Were epidemiologi-cal sources carefully described? | **3)**  Were costs sufficiently disaggre- gated? | **4)**  Were activity data sources carefully described? | **5)**  Were activity data appropriately assessed? | **6)**  Were the sources of all cost values analytically described? | **7)** Were unit costs appropriately valued? | **8)** Were the methods adopted carefully explained? | **9)** Were costs discounted? | **10)** Was the presentation of study results consistent with the methodology of study? | **11)** Were the major assumptions tested in a sensitivity analysis? | Total count of ‘yes’ per study |
| --- | --- | --- | --- | --- | --- | --- | --- | --- | --- | --- | --- | --- |
| Alsuhibani 2022^2^ | Y | Y | NA | Y | Y | NA | NA | Y | NA | Y | N | 6 |
| Amin 2023^3^ | Y | Y | P | Y | Y | Y | U | Y | U | Y | N | 7 |
| Berger 2021^4^ | Y | Y | Y | Y | Y | Y | U | Y | U | Y | Y | 9 |
| Berger 2022^5^ | Y | Y | Y | Y | Y | Y | U | Y | U | Y | Y | 9 |
| Blalock 2023^6^ | Y | Y | NA | Y | Y | NA | NA | Y | NA | Y | N | 6 |
| Blaszczak 2020^7^ | Y | Y | N | Y | Y | Y | U | Y | U | Y | N | 7 |
| Boye 2020^8^ | Y | Y | Y | Y | Y | Y | U | Y | U | Y | Y | 9 |
| Cai 2019^9^ | Y | Y | NA | Y | Y | NA | NA | Y | NA | Y | N | 6 |
| Craig Wood 2021^10^ | Y | Y | NA | Y | Y | NA | NA | Y | NA | Y | N | 6 |
| Cutshall 2021^11^ | Y | Y | NA | Y | Y | NA | NA | Y | NA | Y | N | 6 |
| Dahiya 2023^12^ | Y | Y | N | Y | Y | Y | U | Y | U | Y | N | 7 |
| de Souza de Silva 2019^13^ | Y | Y | P | Y | Y | Y | N | Y | U | Y | N | 7 |
| Ding 2021^14^ | Y | Y | Y | Y | Y | Y | N | Y | U | Y | N | 8 |
| Divino 2021^15^ | Y | Y | Y | Y | Y | Y | NA | Y | U | Y | N | 8 |
| Doane 2023^16^ | Y | Y | Y | Y | Y | Y | U | Y | U | Y | Y | 9 |
| Evans 2023^17^ | Y | Y | Y | Y | Y | Y | NA | Y | U | Y | N | 8 |
| Garvey 2023^18^ | Y | Y | Y | Y | Y | Y | N | Y | U | Y | N | 8 |
| Groves 2023^19^ | Y | Y | N | Y | P | Y | N | Y | U | Y | N | 6 |
| Harris 2019a^20^ | Y | Y | N | Y | Y | N | U | Y | U | Y | N | 6 |
| Harris 2019b^21^ | Y | Y | N | Y | Y | Y | U | Y | U | Y | N | 7 |
| Hoffman 2020^22^ | Y | Y | N | Y | Y | N | U | Y | U | Y | Y | 7 |
| Huckfeldt 2020^23^ | Y | Y | Y | Y | Y | Y | U | Y | U | Y | N | 8 |
| Iyengar 2019^24^ | Y | Y | N | Y | P | P | N | Y | U | Y | N | 5 |
| Johnston 2020^25^ | Y | Y | Y | Y | Y | Y | U | Y | U | Y | Y | 9 |
| Jones 2020^26^ | Y | Y | Y | Y | Y | Y | NA | Y | U | Y | N | 8 |
| Krishnaswami 2019^27^ | Y | Y | NA | Y | Y | NA | NA | Y | NA | Y | N | 6 |
| Kumar 2020^28^ | Y | Y | NA | Y | Y | NA | NA | Y | NA | Y | N | 6 |
| Kumar 2022^29^ | Y | Y | Y | Y | Y | Y | U | Y | U | Y | N | 8 |
| Laliberté 2021^30^ | Y | Y | Y | Y | Y | Y | U | Y | U | Y | Y | 9 |
| Ludhwani 2019^31^ | Y | Y | N | Y | Y | N | U | Y | U | Y | N | 6 |
| MacEwan 2021^32^ | Y | Y | Y | Y | Y | Y | N | Y | U | Y | N | 8 |
| MacEwan 2023^33^ | Y | Y | Y | Y | Y | Y | NA | Y | U | Y | N | 8 |
| McKinney 2021^34^ | Y | Y | NA | Y | Y | NA | NA | Y | NA | Y | N | 6 |
| Mitchell 2023^35^ | Y | Y | P | Y | Y | U | U | Y | U | Y | N | 6 |
| Oladunjoye 2021^36^ | Y | Y | N | Y | Y | Y | U | Y | U | Y | N | 7 |
| Pagidipati 2021^37^ | Y | Y | NA | Y | Y | NA | NA | Y | NA | Y | Y | 7 |
| Patel 2021^38^ | Y | Y | N | Y | Y | Y | U | Y | U | Y | Y | 8 |
| Pearson-Stuttard 2023^39^ | Y | Y | Y | Y | Y | Y | NA | Y | U | Y | N | 8 |
| Perales 2020^40^ | Y | Y | NA | Y | Y | NA | NA | Y | NA | Y | N | 6 |
| Peterson 2019^41^ | Y | Y | Y | Y | Y | Y | U | Y | U | Y | N | 8 |
| Pressman 2023^42^ | Y | Y | NA | Y | Y | NA | NA | Y | NA | Y | N | 6 |
| Rajkumar 2023^43^ | Y | Y | NA | Y | Y | NA | NA | Y | NA | Y | N | 6 |
| Ramasamy 2019^44^ | Y | Y | Y | Y | Y | Y | U | Y | U | Y | N | 8 |
| Ramasamy 2020^45^ | Y | Y | Y | Y | Y | Y | U | Y | U | Y | N | 8 |
| Rives-Sanchez 2020^46^ | Y | Y | NA | Y | Y | NA | NA | Y | NA | Y | N | 6 |
| Rozen 2022^47^ | Y | Y | NA | Y | Y | NA | NA | Y | NA | Y | N | 6 |
| Rozjabek 2020^48^ | Y | Y | P | Y | Y | Y | N | Y | U | Y | N | 7 |
| Schuller 2020^49^ | Y | Y | NA | Y | Y | NA | NA | Y | NA | Y | N | 6 |
| Shaka 2021^50^ | Y | Y | N | Y | Y | Y | U | Y | U | Y | N | 7 |
| Spyropoulos 2019^51^ | Y | Y | Y | Y | Y | Y | U | Y | U | Y | Y | 9 |
| Sreenivasan 2021^52^ | Y | Y | NA | Y | Y | NA | NA | Y | NA | Y | N | 6 |
| Sudat 2023^53^ | Y | Y | NA | Y | Y | NA | NA | Y | NA | Y | N | 6 |
| Surbhi 2022^54^ | Y | Y | NA | Y | Y | NA | NA | Y | NA | Y | N | 6 |
| Sutton 2021^55^ | Y | Y | NA | Y | Y | NA | NA | Y | NA | Y | N | 6 |
| Temkin-Greener 2020^56^ | Y | Y | NA | Y | Y | NA | NA | Y | NA | Y | N | 6 |
| Thorpe 2021^57^ | Y | Y | N | Y | Y | P | NA | Y | U | Y | N | 6 |
| Watkins 2022^58^ | Y | Y | Y | Y | Y | Y | N | Y | U | Y | N | 8 |
| Weir 2021^59^ | Y | Y | Y | Y | Y | Y | U | Y | U | Y | Y | 9 |
| Whitaker 2022^60^ | Y | Y | NA | Y | Y | NA | NA | Y | NA | Y | N | 6 |
| Wu 2019^61^ | Y | Y | Y | Y | Y | Y | U | Y | U | Y | Y | 9 |

HCRU, healthcare resource utilization; N, no; NA, not applicable; P, partial; U, unclear; Y, yes.

# FIGURES

## **FIGURE S1** Number of studies included in SLR, by ORCs.


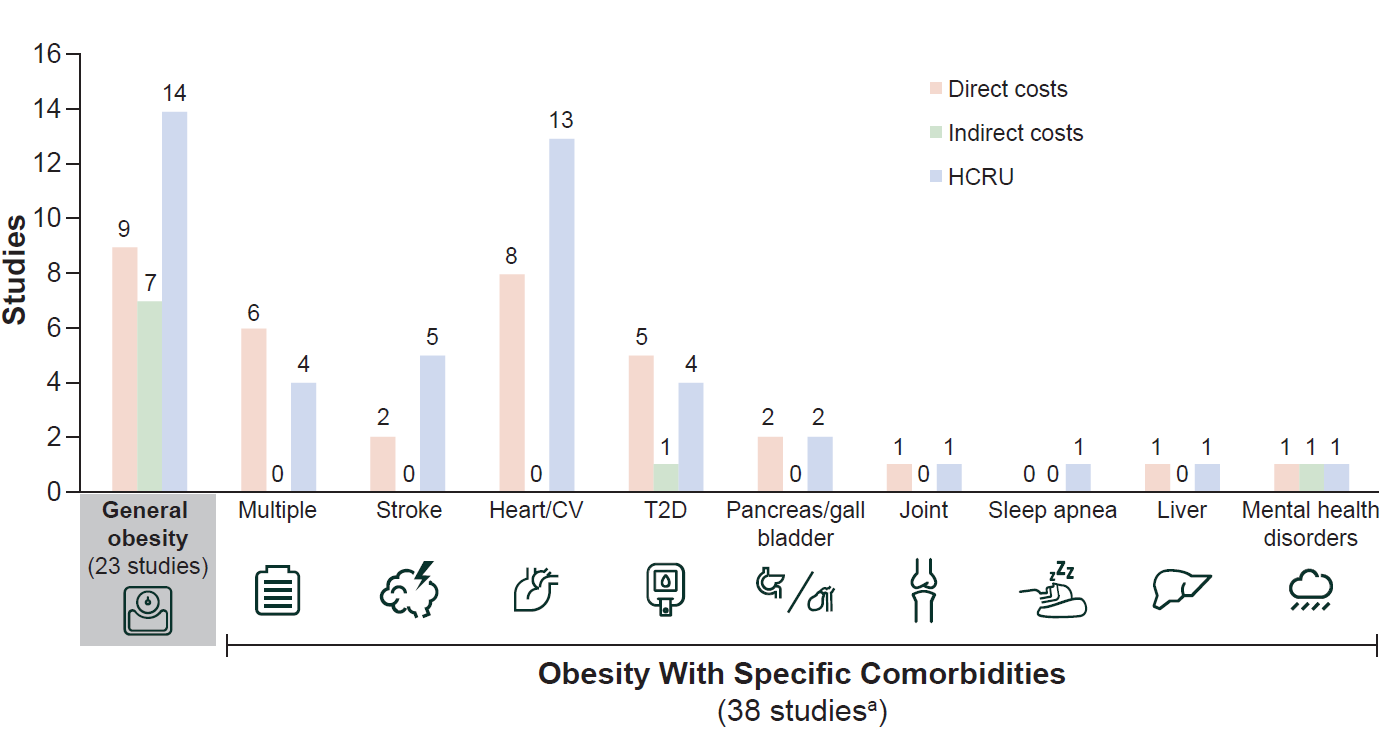


Total number of studies included = 60.

Abbreviations: CV, cardiovascular; HCRU, healthcare resource utilization; ORC, obesity-related complications; SLR, systematic literature review; T2D, type 2 diabetes.

^a^One study (Rozjabek et al., 2020^48^) reported both obesity alone and obesity with T2D and was therefore counted in both categories.

## **FIGURE S2** Hospital LOS in PwO with ORCs.^11,20,31,47,50^


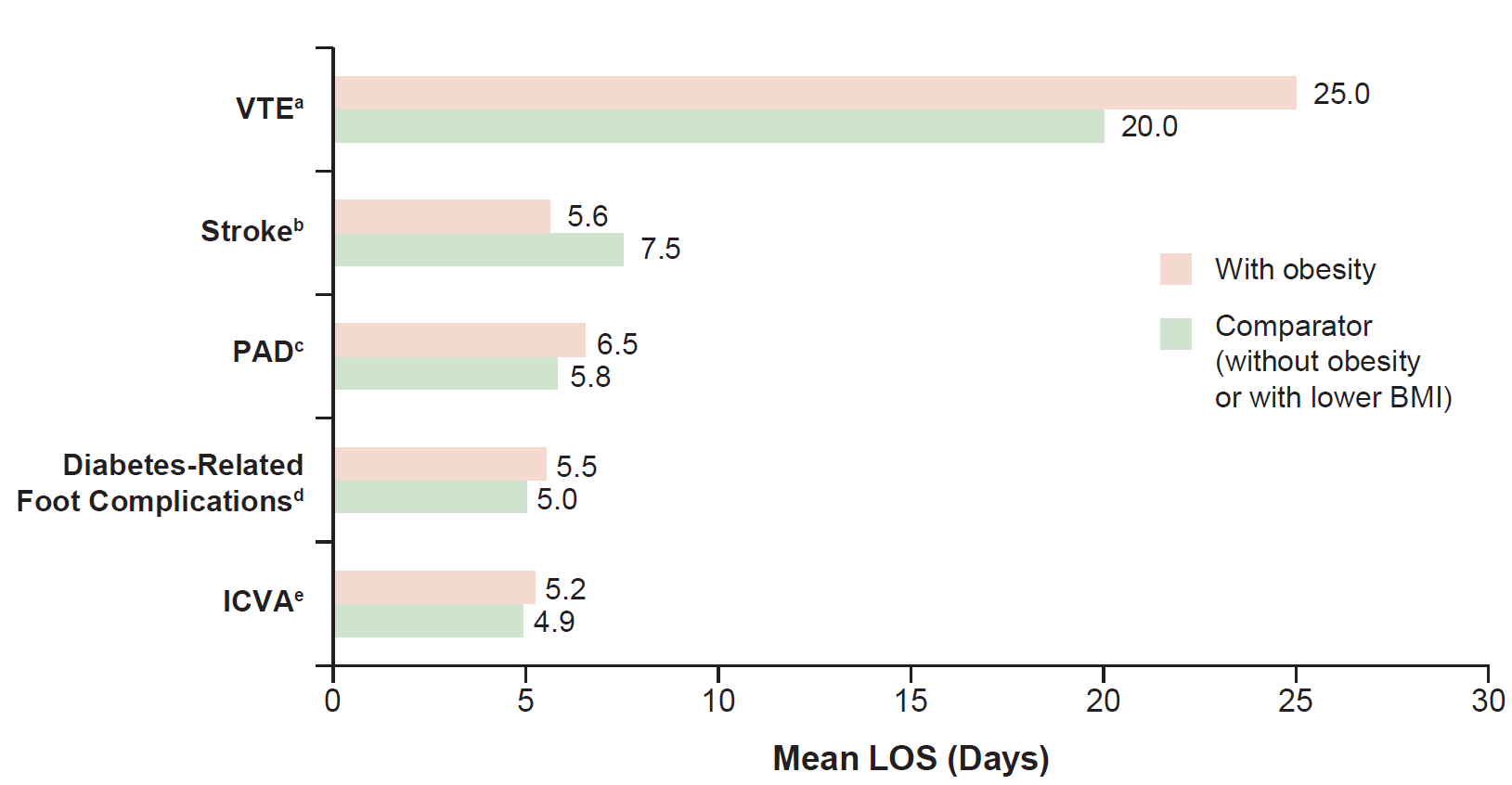


Abbreviations: BMI, body mass index; ICVA, ischemic cerebrovascular accident; LOS, length of stay; ORC, obesity-related complication; PAD, peripheral arterial disease; PwO, people living with obesity; VTE, venous thromboembolism.

^a^BMI ≥40 vs 30–34.9 kg/m^2^.
^b^BMI ≥40 vs 20–25 kg/m^2^; includes all types of stroke.
^c^BMI >25 vs <25 kg/m^2^.
^d^BMI ≥40 vs <40 kg/m^2^.
^e^BMI ≥30 vs <30 kg/m^2^; includes only ischemic stroke.

#

# REFERENCES

1. Molinier L, Bauvin E, Combescure C, et al. Methodological considerations in cost of prostate cancer studies: a systematic review. *Value Health.* 2008;11(5):878–885.

2. Alsuhibani A, Alrasheed M, Gari M, Hincapie AL, Guo JJ. Descriptive analysis of reported adverse events associated with anti-obesity medications using FDA Adverse Event Reporting System (FAERS) databases 2013-2020. *Int J Clin Pharm.* 2022;44(1):172–179.

3. Amin A, Kartashov A, Ngai W, Steele K, Rosenthal N. Effectiveness, safety, and costs of thromboprophylaxis with enoxaparin or unfractionated heparin in inpatients with obesity. *Front Cardiovasc Med.* 2023;10:1163684.

4. Berger JS, Laliberte F, Kharat A, et al. Healthcare resource utilization and costs of rivaroxaban versus warfarin among non-valvular atrial fibrillation (NVAF) patients with obesity in a US population. *J Med Econ.* 2021;24(1):550–562.

5. Berger JS, Laliberte F, Kharat A, et al. Effectiveness, safety, and healthcare costs associated with rivaroxaban versus warfarin among venous thromboembolism patients with obesity: a real-world study in the United States. *J Thromb Thrombolysis.* 2022;54(3):438–448.

6. Blalock DV, Pura JA, Stechuchak KM, et al. BMI trends for veterans up to 10 years after VA enrollment following military discharge. *J Gen Intern Med.* 2023;38(6):1423–1430.

7. Blaszczak AM, Krishna SG, Hart PA, et al. Class III obesity rather than metabolic syndrome impacts clinical outcomes of acute pancreatitis: a propensity score weighted analysis. *Pancreatology.* 2020;20(7):1287–1295.

8. Boye KS, Lage MJ, Terrell K. Healthcare outcomes for patients with type 2 diabetes with and without comorbid obesity. *J Diabetes Complications.* 2020;34(12):107730.

9. Cai S, Wang S, Mukamel DB, Caprio T, Temkin-Greener H. Hospital readmissions among post-acute nursing home residents: does obesity matter? *J Am Med Dir Assoc.* 2019;20(10):1274–1279.e1274.

10. Craig Wood G, Bailey-Davis L, Benotti P, et al. Effects of sustained weight loss on outcomes associated with obesity comorbidities and healthcare resource utilization. *PLoS One.* 2021;16(11):e0258545.

11. Cutshall BT, Tatara AW, Upadhyay N, Adeola M, Putney D, Ruegger M. Evaluating time to in-hospital venous thromboembolism in obese patients. *J Pharm Pract.* 2021;34(2):190–198.

12. Dahiya DS, Sharma NR, Perisetti A, et al. The influence of obesity on acute pancreatitis hospitalizations: does body mass index matter? *Pancreas.* 2023;52(3):E171–E178.

13. de Souza de Silva CG, Kokkinos P, Doom R, et al. Association between cardiorespiratory fitness, obesity, and health care costs: the Veterans Exercise Testing Study. *Int J Obes (Lond).* 2019;43(11):2225–2232.

14. Ding Y, Fan X, Blanchette CM, Gabriel Smolarz B, Weng W, Ramasamy A. Economic value of nonsurgical weight loss in adults with obesity. *J Manag Care Spec Pharm.* 2021;27(1):37–50.

15. Divino V, Ramasamy A, Anupindi VR, et al. Complication-specific direct medical costs by body mass index for 13 obesity-related complications: a retrospective database study. *J Manag Care Spec Pharm.* 2021;27(2):210–222.

16. Doane MJ, Thompson J, Jauregui A, Gasper S, Csoboth C. Clinical, economic, and humanistic outcomes associated with obesity among people with bipolar I disorder in the United States: analysis of national health and wellness survey data. *Clinicoecon Outcomes Res.* 2023;15:681–689.

17. Evans M, Anupindi VR, DeKoven M, et al. Eight-year trends in obesity-related complications and health care cost progression in a US population with obesity: a retrospective cohort study. *Diabetes Obes Metab.* 2023;25(2):536–544.

18. Garvey WT, Cheng M, Ramasamy A, et al. Clinical and cost benefits of anti-obesity medication for US veterans participating in the MOVE! Weight Management Program. *Popul Health Manag.* 2023;26(1):72–82.

19. Groves J, Wilcox V. The impact of overweight and obesity on unemployment duration among young American workers. *Econ Hum Biol.* 2023;51:101280.

20. Harris CM, Abougergi MS, Wright S. Clinical outcomes among morbidly obese patients hospitalized with diabetic foot complications. *Clin Obes.* 2019;9(1):e12285.

21. Harris CM, Albaeni A, Wright S, Norris KC. Obesity as a risk factor among hospitalized patients with infective endocarditis. *Open Forum Infect Dis.* 2019;6(10):ofz390.

22. Hoffman H, Jalal MS, Furst T, Chin LS. The obesity paradox in spontaneous intracerebral hemorrhage: results from a retrospective analysis of the nationwide inpatient sample. *Neurocrit Care.* 2020;32(3):765–774.

23. Huckfeldt PJ, Frenier C, Pajewski NM, et al. Associations of intensive lifestyle intervention in type 2 diabetes with health care use, spending, and disability: an ancillary study of the Look AHEAD Study. *JAMA Netw Open.* 2020;3(11):E2025488.

24. Iyengar JJ, Miller NM, Ajluni N, et al. Impact of a structured weight management program on worker productivity. *J Occup Environ Med.* 2019;61(2):148–152.

25. Johnston SS, Ammann E, Scamuffa R, et al. Association of body mass index and osteoarthritis with healthcare expenditures and utilization. *Obes Sci Pract.* 2020;6(2):139–151.

26. Jones D, Martinez-Amezcua P, Pandian V. Utilization of resources by patients who are morbid and super obese admitted to a tertiary care center. *J Patient Saf.* 2020;16(2):143–148.

27. Krishnaswami A, Sidney S, Sorel M, Smith W, Ashok R. Temporal changes in health care utilization among participants of a medically supervised weight management program. *Perm J.* 2019;23:18–134.

28. Kumar SI, Doo K, Sottilo-Brammeier J, Lane C, Liebler JM. Super obesity in the medical intensive care unit. *J Intens Care Med.* 2020;35(5):478–484.

29. Kumar V, Encinosa W. Revisiting the obesity paradox in health care expenditures among adults with diabetes. *Clin Diabetes.* 2022;40(2):185–195.

30. Laliberté F, Ashton V, Kharat A, et al. Economic burden of rivaroxaban and warfarin among nonvalvular atrial fibrillation patients with obesity and polypharmacy. *J Comp Eff Res.* 2021;10(16):1235–1250.

31. Ludhwani D, Wu J. Obesity paradox in peripheral arterial disease: results of a propensity match analysis from the national inpatient sample. *Cureus.* 2019;11(5):e4704.

32. MacEwan J, Kan H, Chiu K, Poon JL, Shinde S, Ahmad NN. Antiobesity medication use among overweight and obese adults in the United States: 2015-2018. *Endocr Pract.* 2021;27(11):1139–1148.

33. MacEwan JP, Chiu K, Ahmad NN, et al. Clinical, economic, and health-related quality of life outcomes in patients with overweight or obesity in the United States: 2016-2018. *Obes Sci Pract.* 2023;10(1):e726.

34. McKinney AL, Dailey LM, McMillen JC, Rowe AS. Impact of obesity on warfarin reversal with fixed-dose factor VIII inhibitor bypassing activity (aPCC). *Ann Pharmacother.* 2021;55(7):856–862.

35. Mitchell ES, Fabry A, Ho AS, et al. The impact of a digital weight loss intervention on health care resource utilization and costs compared between users and nonusers with overweight and obesity: retrospective analysis study. *JMIR Mhealth and Uhealth.* 2023;11:e47473.

36. Oladunjoye O, Oladunjoye AO, Dhital R, et al. A retrospective study of hospitalizations in the USA: proportion of hospitalizations with non-alcoholic fatty liver disease in non-obese population. *Cureus.* 2021;13(9):e17869.

37. Pagidipati NJ, Phelan M, Page C, et al. The importance of weight stabilization amongst those with overweight or obesity: results from a large health care system. *Prev Med Rep.* 2021;24:101615.

38. Patel N, Vyas R, Srinivasan S, Mital D. Hospitalisation characteristics of metabolic syndrome patients. *Int J Med Eng Inform.* 2021;13(6):449–460.

39. Pearson-Stuttard J, Banerji T, Capucci S, et al. Real-world costs of obesity-related complications over eight years: a US retrospective cohort study in 28,500 individuals. *Int J Obes.* 2023;47(12):1239–1246.

40. Perales IJ, San Agustin K, DeAngelo J, Campbell AM. Rivaroxaban versus warfarin for stroke prevention and venous thromboembolism treatment in extreme obesity and high body weight. *Ann Pharmacother.* 2020;54(4):344–350.

41. Peterson ED, Ashton V, Chen YW, Wu B, Spyropoulos AC. Comparative effectiveness, safety, and costs of rivaroxaban and warfarin among morbidly obese patients with atrial fibrillation. *Am Heart J.* 2019;212:113–119.

42. Pressman A, Jones JB, Xu X, et al. Characterizing obesity in a large health care delivery system. *Am J Manag Care.* 2023;29(11):558–564.

43. Rajkumar S, Davidson E, Bell M, et al. Effect of telehealth-based versus in-person nutritional and exercise intervention on type II diabetes mellitus improvement and efficiency of human resources utilization in patients with obesity. *Obes Sci Pract.* 2023;9(5):468–476.

44. Ramasamy A, Laliberte F, Aktavoukian SA, et al. Direct and indirect cost of obesity among the privately insured in the United States: a focus on the impact by type of industry. *J Occup Environ Med.* 2019;61(11):877–886.

45. Ramasamy A, Laliberte F, Aktavoukian SA, et al. Direct, absenteeism, and disability cost burden of obesity among privately insured employees: a comparison of healthcare industry versus other major industries in the United States. *J Occup Environ Med.* 2020;62(2):98–107.

46. Rives-Sanchez M, Quintos A, Prillaman B, et al. Sleep disordered breathing in hospitalized African-Americans. *J Natl Med Assoc.* 2020;112(3):262–267.

47. Rozen G, Elbaz-Greener G, Margolis G, et al. The obesity paradox in real-world nation-wide cohort of patients admitted for a stroke in the U.S. *J Clin Med.* 2022;11(6):1678.

48. Rozjabek H, Fastenau J, Laprade A, Sternbach N. Adult obesity and health-related quality of life, patient activation, work productivity, and weight loss behaviors in the United States. *Diabetes Metab Syndr Obes.* 2020;13:2049–2055.

49. Schuller KA. Is obesity a risk factor for readmission after acute myocardial infarction? *J Healthc Qual Res.* 2020;35(1):4–11.

50. Shaka H, El-Amir Z, Wani F, Dahiya DS, Velazquez GR, Kichloo A. The paradox: ischemic cerebrovascular accidents and obesity - a retrospective nationwide inpatient study. *Obes Med.* 2021;23:100339.

51. Spyropoulos AC, Ashton V, Chen YW, Wu B, Peterson ED. Rivaroxaban versus warfarin treatment among morbidly obese patients with venous thromboembolism: comparative effectiveness, safety, and costs. *Thromb Res.* 2019;182:159–166.

52. Sreenivasan J, Khan MS, Sharedalal P, et al. Obesity and outcomes following cardiogenic shock requiring acute mechanical circulatory support. *Circ Heart Fail.* 2021;14(3):E007937.

53. Sudat SEK, Huang Q, Szwerinski N, Romanelli RJ, Azar KMJ. Changes in healthcare utilization after lifestyle intervention for weight loss. *Am J Prev Med.* 2024;66(4):619–626.

54. Surbhi S, Chen M, Shuvo SA, et al. Effect of continuity of care on emergency department and hospital visits for obesity-associated chronic conditions: a federated cohort meta-analysis. *J Natl Med Assoc.* 2022;114(5):525–533.

55. Sutton LH, Tellor BR, Pope HE, Riney JN, Weaver KL. Evaluation of time to therapeutic anticoagulation and associated outcomes in critically ill, obese patients with pulmonary embolism receiving unfractionated heparin. *J Pharm Pract.* 2021;34(3):438–444.

56. Temkin-Greener H, Wang S, Caprio T, Mukamel DB, Cai S. Obesity among nursing home residents: association with potentially avoidable hospitalizations. *J Am Med Dir Assoc.* 2020;21(9):1331–1335.e1331.

57. Thorpe K, Toles A, Shah B, Schneider J, Bravata DM. Weight loss-associated decreases in medical care expenditures for commercially insured patients with chronic conditions. *J Occup Environ Med.* 2021;63(10):847–851.

58. Watkins S, Toliver JC, Kim N, Whitmire S, Garvey WT. Economic outcomes of antiobesity medication use among adults in the United States: a retrospective cohort study. *J Manag Care Spec Pharm.* 2022;28(10):1066–1079.

59. Weir MR, Chen YW, He J, Bookhart B, Campbell A, Ashton V. Healthcare resource utilization and costs of rivaroxaban versus warfarin among nonvalvular atrial fibrillation patients with obesity and diabetes. *Diabetes Ther.* 2021;12(12):3167–3186.

60. Whitaker C, McKinney A, Bollig R, Hieb N, Roberts RF, Rowe AS. Incidence of thrombotic complications related to weight-based dosing of activated prothrombin complex concentrate (aPCC) for reversal of apixaban and rivaroxaban in obese patients. *J Thromb Thrombolysis.* 2022;53(4):861–867.

61. Wu J, Davis-Ajami ML, Lu ZK. Real-world impact of ongoing regular exercise in overweight and obese US adults with diabetes on health care utilization and expenses. *Prim Care Diabetes.* 2019;13(5):430–440.

1. * Corresponding author at [Jaime.Almandoz@UTSouthwestern.edu](mailto:Jaime.Almandoz@UTSouthwestern.edu)
   Internal Medicine Subspecialties Clinic, West Campus Building 3, 8th Floor, 2001 Inwood Rd., Dallas, TX 75390-9255 [↑](#footnote-ref-1)
