# Supplementary figures and images for: Healthcare Resource Utilisation and Cost of Obesity and Related Complications in the United States: A Systematic Literature Review
Source: Diabetes Obes Metab. 2026 Mar 9;28(5):4121–36. doi: 10.1111/dom.70602 (PMC13071262; doi:10.1111/dom.70602)

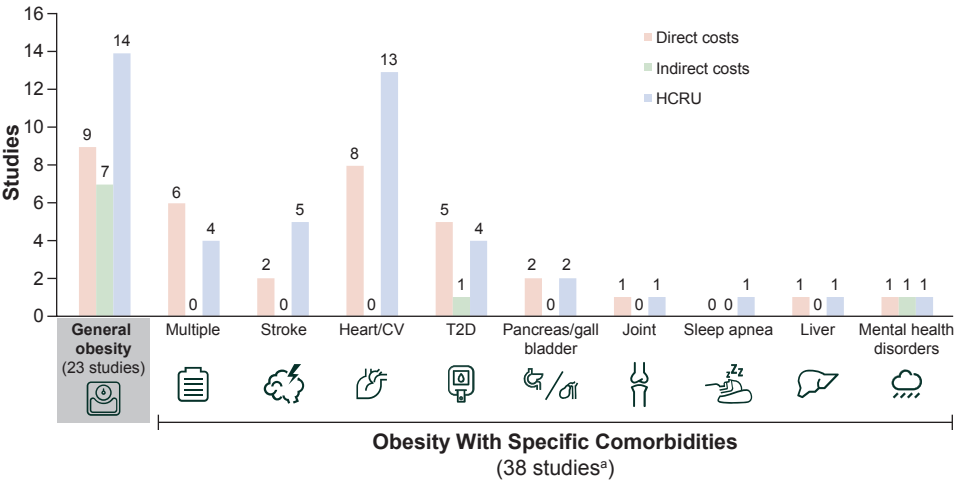

Supplement: Supplementary file 2 — Figure S1: dom70602‐sup‐0002‐Figure S1.pdf. [file DOM-28-4121-s001.pdf]

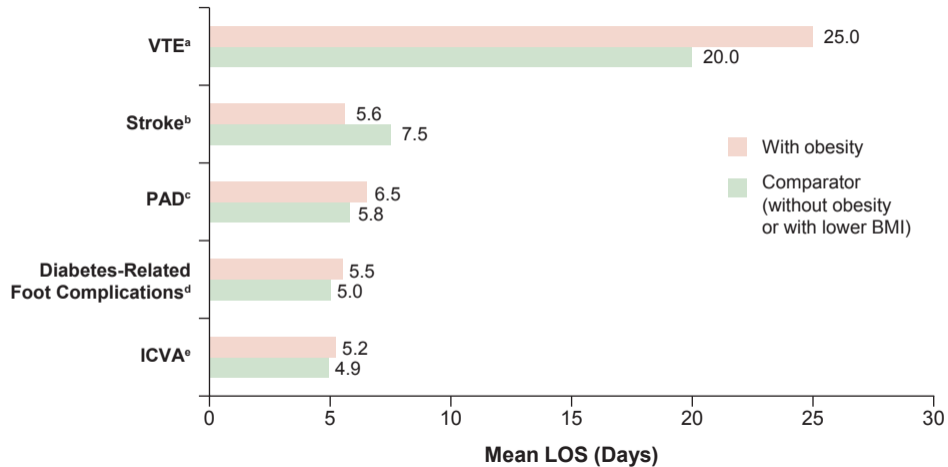

Supplement: Supplementary file 3 — Figure S2: dom70602‐sup‐0003‐Figure S2.pdf. [file DOM-28-4121-s002.pdf]
